# A systematic review and meta-analysis of clinical trials on saffron (*Crocus sativus*) effectiveness and safety on erectile dysfunction and semen parameters

**Published:** 2018

**Authors:** Nahid Maleki-saghooni, Khadijeh Mirzaeii, Hossein Hosseinzadeh, Ramin Sadeghi, Morvarid Irani

**Affiliations:** 1 *Student Research Committee, Department of Midwifery, School of Nursing and Midwifery, Mashhad University of Medical Sciences, Mashhad, Iran *; 2 *Faculty of Nursing and Midwifery, Mashhad University of Medical Sciences, Mashhad, Iran*; 3 *Pharmaceutical Research Center, Department of Pharmacodynamics and Toxicology, School of Pharmacy, Mashhad University of Medical Sciences, Mashhad, Iran*; 4 *Nuclear Medicine Research Center, Mashhad University of Medical Sciences, Mashhad, Iran*

**Keywords:** Saffron (Crocus sativus), Erectile dysfunction, Semen parameters, Male infertility, Meta-analysis

## Abstract

**Objective:**

We performed this systematic review and meta-analysis study to determine saffron (*Crocus sativus*) effectiveness and safety in male infertility problems.

**Materials and Methods:**

The databases PubMed, Scopus, Cochrane, Google Scholar, SID, IranMedex and Magiran until July 2016 and reference section of relevant articles, were searched to find both English and Persian clinical trials on male infertility issues that used saffron as medical treatment. Also, the quality of these trials was evaluated by Oxford Center for Evidence Based Medicine checklist. A total of six trials was ultimately included. All statistical analyses were done by Comprehensive Meta-analysis (CMA) Version 2.

**Results:**

Only in one study conducted on sperm parameters, the mean percentage of sperm with normal morphology (p<0.001) and sperm motility (p<0.001) were increased. Quantitative analysis showed that saffron had a significantly positive effect on all dimensions of Erectile Function questionnaire (MD for Erectile function=5.36(p=0.00), Orgasmic function=1.12 (p=0.007), Overall satisfaction=1.23 (p=0.005), Satisfaction with intercourse=2.18 (p=0.00) and Sexual desire=0.78 (p=0.00), Fixed effects model using 3 trials). The result of subgroup analysis based on dimensions of Erectile Function questionnaire showed statistically significant differences among subgroups (p=0.00).

**Conclusion:**

saffron has a positive effect on erectile dysfunction. However, our study showed contradictory results about semen parameters. So, interpretation of results is limited because of methodological flaws of the included studies, erectile dysfunction status and a large heterogeneity among them. Further trials are still needed to confirm the current findings.

## Introduction

One of male infertility problems is infertility that is defined as the failure to get pregnant after one year of regular, unprotected intercourse. Approximately 15% of couples are infertile, and in half of these cases, a male factor is involved (Hamada et al., 2012 ▶, Mosher, 1988). Infertility resulting in permanent childlessness is exceptionally troublesome for couples (Downey et al., 1989 ▶, Whiteford and Gonzalez, 1995 ▶). It is stated that one out of twenty men in the general population is infertile. Different medicines such as clomiphene citrate and cancer prevention agents are utilized to help these patients. However, none of them has resulted in a complete treatment (Isidori et al., 2006 ▶, Kumar et al., 2006 ▶). Hence, other alternative agents are required to overcome this problem. Recent studies have explored the effect of reactive oxygen species (ROS) on sperm. Increased levels of ROS have been shown to be related with diminished motility of the spermatozoa and increased DNA damage, and may even induce germ cell apoptosis (Irani et al.,2017 ▶,Agarwal and Said, 2005 ▶, Wang et al., 2003 ▶). In fact, studies have shown that more than 40% of infertile men have increased levels of ROS in their seminal plasma (Zini et al., 1993 ▶). Therefore, several studies have concentrated on the usage of antioxidant agents in order to prevent ROS effects on sperm metabolism, motility, morphology, and as a result, fertilizing capacity. Erectile dysfunction (ED) is the most common sexual dysfunction among men. It is characterized as a persistent or recurrent failure to maintain erection for a satisfactory intercourse (Heidelbaugh, 2010 ▶, Smith et al., 2008 ▶). ED affects more than 150 million males throughout the world (Porst et al., 2003 ▶). This dysfunction is known to influence physical, psychological, and social health of parents and the families as well as their quality of life (Hatzimouratidis et al., 2010 ▶). In a minority of cases, phosphodiesterase 5-inhibitors, like sildenafil, tadalafil and vardenafil resolve ED; However due to various reasons such as side-effects, cost and drug interactions, many men stop using them and seek other treatments. Crocus sativus (saffron) is a perpetual herb of the Iridaceae family with anti-oxidative prosperities (Pham et al., 2000 ▶, Botsoglou et al., 2005 ▶, Kanakis et al., 2007 ▶). It generally grows in Iran, India, Greece, Spain, and France and its dried red stigma is utilized as a food spice. Furthermore, saffron has been used in folk medicine (Basker and Negbi, 1983 ▶, Rios et al., 1996 ▶). Interest in the use of saffron as a treatment, is raising because of its considerable anti-oxidative properties (Abdullaev, 2002 ▶, Verma and Bordia, 1998 ▶) and pharmacological activities such as anti-catarrhal, antidepressant, carminative, anticonvulsant, antispasmodic, emmenagogue, and particularly for antitumor and sexual enhancing effects (Abdullaev and Espinosa-Aguirre, 2004 ▶, Hosseinzadeh et al., 2008 ▶). In the ancient medicine of countries such as India, Spain, and China, saffron has been utilized to treat infertility and impotence (Abdullaev and Espinosa-Aguirre, 2004 ▶, Mollazadeh et al., 2015, Hosseinzadeh and Nassiri Asl, 2013 ▶, Chatterjee et al., 2005 ▶); So, it has been perceived as a sexual stimulant (Mousavi and Bathaie, 2011 ▶, De Liz and Strauss, 2005 ▶). There is a growing interest in saffron as a treatment of erectile dysfunction (Shamsa et al., 2009 ▶, Safarinejad et al., 2010 ▶, Mohammadzadeh-Moghadam et al., 2015 ▶, Modabbernia et al., 2012 ▶) and men infertility (Safarinejad et al., 2011 ▶, Heidary et al., 2008 ▶). Accordingly, we prepared this systematic review and meta-analysis study to investigate clinical trials on saffron effectiveness and safety in male infertility problems.

## Materials and Methods

**Data sources and search strategy**

 A systematic literature review was done using the electronic databases namely, PubMed, Scopus, Cochrane, and Google Scholar as well as Persian databases such as SID, Iran Medex, and Magiran, using related keywords to find articles published until July 2016. Our keywords were (saffron OR *Crocus sativus* Linn. OR Safranal OR Crocin) AND (Semen Parameters) OR (erectile dysfunction) OR (Sexual Dysfunction) OR (male fertility problems) in Title, Abstract, or Keywords. All of aforementioned databases were searched for published clinical trials in both English and Persian languages. Our search resulted in finding duplicate articles, or articles which were inconsequential to the investigation, which were not included in this review. Moreover, reference section of relevant trials, systematic reviews and meta-analyses were manually checked to recognize related trials missed by electronic databases search. In the process of finding articles, one of the investigators assessed both the Title and Abstract of the articles to decide its suitability for incorporation. 

The selected articles needed to meet the following criteria to be included in this review:

1. Study subjects must be men receiving saffron as a treatment.

2. The control groups received placebo or other medicines.

3. Only randomized controlled studies and clinical trials written in in both English and Persian languages published until July 2016 were included. 

1. Outcomes that were evaluated were erectile dysfunction and semen parameters. 

**Figure 1:**
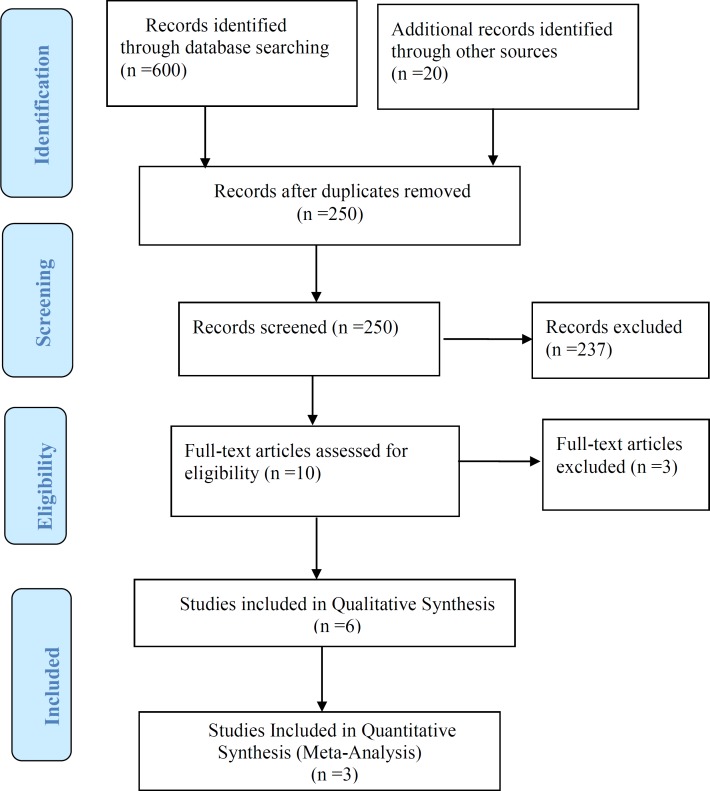
PRISMA Flowchart of the study selection process

The procedure of the search and selection of RCTs are shown in Figure 1.

**Study selection and data extraction**

The study selection and data extraction were done by two independent reviewers. According to search results, authors scanned Results, Abstracts and relevant records. Full articles of all potentially relevant trials were retrieved. All retrieved studies were scrutinized to check for multiple publications of the same trials. For each study, we extracted the accompanying data according to a pre-defined checklist: first author, year, country, study design, participants, intervention, comparisons, dropout, tools, outcome and quality of trials. Data were evaluated by two reviewers and variations were settled following discussions with a third researcher. Overall, there was a complete agreement between the two reviewers. The outlined attributes of the included studies are shown in Table 1.

**Evaluation of the quality of the included studies**

The quality of the included studies was assessed using Oxford Center for Evidence Based Medicine checklist for RCTs (see Table 2).

Criteria for surveying the quality of studies:

A: Was the assignment of patients to treatments randomized?

B: Were the groups similar at the start of the trial?

C: Aside from the assigned treatment, were groups treated equally?

D: Were all patients who entered the trial accounted for at its conclusion? – And were they analyzed in the groups to which they were randomly assigned? (1: Losses to follow-up and 2: intention-to-treat).

E: Were measures objective and were the patients and clinicians kept “blind” to the treatment given?

F: What were the outcomes? (Howick et al., 2011 ▶)

**Quality evaluation of the included studies**

The quality of the included studies was assessed by Oxford Center for Evidence Based Medicine checklist for RCTs (Howick et al., 2011 ▶). This tool is designed that assess Internal Validity: consisting of six general questions about the way of patients assignment, similarity and matching of groups, equality of allocated treatment, losses to follow-up and intention-to-treat analysis, blindness and effect size which was answered with three options Yes , No and Unclear.

**Statistical analyses**

We interpreted the results utilizing effect sizes, for heterogeneity evaluation, Cochrane Q test (p<0.05 as statistically significant) and I^2^ index.

Random effects models of analysis were used if heterogeneity was not detected (p=0.462; I^2^=0%). The pooled estimates of Studies were calculated. The last was utilized to assess if the variance among studies is likely to be real and is not because of sampling errors. All statistical analyses were done using Comprehensive Meta-analysis Version 2 (Biostat, Englewood, NJ, USA).

## Results

A total of six RCTs met the inclusion criteria and were incorporated into the meta-analysis (see Table 1). 

**The impact of saffron on Semen Parameters**

Two trials (Heidary et al. 2008 ▶, Safarinejad et al. 2011 ▶) examined the impact of saffron on semen parameters. One trial by Heidary et al., utilizing paired t-test, found that the mean percentage of sperm with normal morphology was 26.50 ± 6.44% before the treatment which increased to 33.90 ± 10.45%, thereafter (p<.001). Significant increases were likewise found in the percentages of class A to class C morphology of the sperm. The mean percentage of sperm with Class A motility was 5.32 ± 4.57% before and 11.77% ± 6.07% after the treatment (p<0.001). Class B and C motilities were initially 10.09 ± 4.20% and 19.79 ± 9.11% which increased to 17.92 ± 6.50% (p<0.001) and 25.35 ± 10.22% (p<0.001), respectively. Overall, 6.4%, 7.8%, and 5.6% increases were detected in the percentage of sperm with class A, B, and C motility, respectively. They could not recognize a significant increase in terms of sperm count following saffron therapy; the mean sperm count was initially 43.45 ± 31.29 × 10^6^/mL which increased to 44.92 ± 28.36× 10^6^/mL (p=0.30).

In the second trial by Safarinejad et al. saffron administration did not result in beneﬁcial impacts and no statistically signiﬁcant improvements were seen in either group in any of the semen parameters namely, sperm density, morphology and motility (for all cases p=0.1). The mean sperm count, sperm concentration, sperm motility and sperm morphology remained relatively constant in each of the treatment groups throughout the study period (for all cases p=0.1). These semen parameters were close to those obtained at baseline. According to analysis of variance with multiple comparisons, treatment with saffron did not cause an increase in seminal plasma catalase-like activity (p=0.1). In other words, saffron administration did not enhance total seminal plasma antioxidant capacity, compared with baseline (p=0.1) and placebo subjects (p=0.1) (Heidary et al., 2008 ▶, Safarinejad et al., 2011 ▶).

**The impact of saffron on Erectile Dysfunction (International Index of Erectile Function Questionnaire and Its Dimensions)**

**Figure 2:**
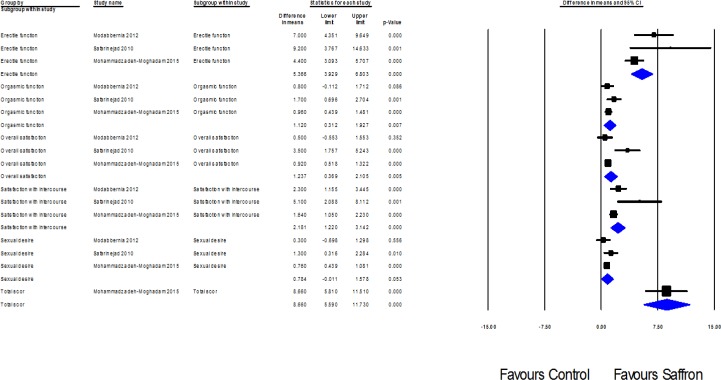
Effects of saffron on Erectile Dysfunction. The horizontal lines denote the 95% CI, ■ point estimate (size of the square corresponds to its weight); ♦, combined overall effect of treatment

Four trials (Shamsa et al., 2009 ▶, Modabbernia et al. 2012 ▶, Safarinejad et al., 2010 ▶, Mohammadzadeh-Moghadam et al., 2015 ▶) assessed the effect of saffron on Erectile Dysfunction. Of these four studies, trial by Shamsa et al. was not included in our quantitative analysis because of the absence of a placebo group. Pooled MD for Erectile function 5.36; 95% CI: 3.92 to 6.80; p=0.00; heterogeneity p=0.07; I2=62%; Pooled MD for Orgasmic function 1.12; 95% CI: 0.31 to 1.92; p=0.007; heterogeneity p=0.36; I2=66%; Pooled MD for Overall satisfaction 1.23; 95% CI: 0.36 to 2.10; p=0.005; heterogeneity p=0.01; I2=77%; Pooled MD for Satisfaction with intercourse 2.18; 95% CI: 1.22 to 3.14; p=0.00; heterogeneity p=0.06; I2=63%; Pooled MD for Sexual desire 0.78; 95% CI: -0.01 to 1.57; p=0.00; heterogeneity p=0.37; I2=0%; 384 men; Fixed effects model; 3 trials, which was statistically significant in all cases namely, Erectile function, Orgasmic function, Overall satisfaction, Satisfaction with intercourse and Sexual desire (Shamsa et al., 2009 ▶, Safarinejad et al., 2010 ▶, Mohammadzadeh-Moghadam et al., 2015 ▶, Modabbernia et al., 2012 ▶). The forest plot is shown in Figure 2. 

**Table 1:** Characteristics of the 6 trials included in our systematic review

**Author year Country**	**Design**	**Age (Y)**	**Patient**	**Intervention mg**	**control**	**Baseline comparability**	**Dropouts** ** (%)**	**Tools**	**blinding method**	**Outcome(s)**	**Adverse events**	**Quality indicators:** **Assignment** **-** **groups similar** **-** ** groups treated equally** **- Losses to follow-up-** ** blindness** **-** ** What were the results**
**Shamsa et al. 2009 Iran**	open clinical trial (one group	26 to 62	male with erectile dysfunction n=20	saffron tablet 200 mg each morning for ten days	-	yes	0	international index of erectile function (ILEF-15) nocturnal penile tumescence (NPT) test	Not reported	There was a statistically significant improvement in tip rigidity and tip tumescence as well as base rigidity and base tumescence. ILEF-15 total scores were significantly higher in patients after saffron treatment (p<0.001)	Not reported	-/-/+/+/-/+
**Heidary et al. 2008 Iran**	clinical trial (one group)	21 to 48 years	infertile men n=52	saffron solved in milk 50 mg for 3 month	-	yes	0	History- urologic examination- semen analysis	Not reported	Saffron, as an antioxidant, is positively effective on sperm morphology and motility in infertile men, while it does not increase sperm Count.	Not reported	-/-/+/+/-/+
**Modabbernia** ** et al. 2012 Iran**	RCT	18 to 45	married male patients n=15	saffron 15 mg twice per day for 4-week	Placebo n=15	yes	20	International Index of Erectile Function Hamilton depression rating scale (HDRS)	double-blind	saffron resulted in significantly greater improvement in erectile function (P<0.001) and intercourse satisfaction domains (P00.001), and total scores (P<0.001) than the placebo group	Frequency of side effects were similar between the two groups Daytime drowsiness Nausea, Decreased appetite, Dry mouth Nervousness Restlessness, Morning drowsiness, Increased appetite from 7-20%	+/+/+/+/+/+
**Safarinejad et al. 2010 Iran**	RCT (Crossover study)	18 to 60	men with erectile dysfunction n=155	capsule of saffron 15 mg,	men with erectile dysfunction n=152	yes	10	International Index of Erectile Function (IIEF) the Erectile Dysfunction Inventory of Treatment Satisfaction (EDITS) Global Efficacy Questionnaire (GEQ)	Not reported	No significant improvements were observed with regard to the IIEF sexual function domains; SEP questions and EDITS scores with saffron administration. findings do not support a beneficial effect of saffron administration in men with ED	Adverse effects related to treatment were noted in 20.8% of patients taking sildenafil and 4.0% of patients taking saffron (P<0.001).In patients taking saffron: headache 2.9% flushing 2.3% Nausea 2.3% dyspepsia 1.7% Diarrhea 1.2% Most adverse events were mild to moderate	+/+/+/+/-/+
**Safarinejad et al. 2011 Iran**	RCT	24 to 41 years	infertile men n=114	saffron 60 mg/day for 26 weeks	placebo capsules N=116	yes	11	Genetic analyses- semen analyses- Doppler ultrasound Seminal plasma antioxidant status by nitro-blue tetrazolium (NBT)	double-blind	Saffron administration did not result in beneficial effects in infertile men. At the end of the study no statistically significant improvements were observed in either group in any of the studied semen parameters (p = 0.1). Saffron administration did not improve total seminal plasma antioxidant capacity, compared with baseline (p = 0.1) and placebo subjects (p = 0.1).	Most adverse events were mild to moderate in nature. In patients taking saffron: Decreased platelet count 62.3%, Decreased leukocyte count 60% Decreased red blood cell count (1000/mL) 55.4% Headache 11.5% Nausea 9.2% Sedation 7.7% Hypomania 7.7% Decreased and increased appetite 13.1%	+/+/+/+/+/+
**Mohammadzadeh- Moghadam et al. 2015 Iran**	RCT (parallel-group)	40 to 76	diabetic men N=25	topical saffron for 1 month	placebo N=25	yes	47	International Index of Erectile Function (IIEF)	double-blind	Compared to placebo, the prepared saffron gel could significantly improve erectile dysfunction in diabetic patients (P < .001). This preliminary evidence suggests that saffron can be considered as a treatment option for diabetic men with erectile dysfunction.	Not reported	+/+/+/+/-/+

**Figure 3:**
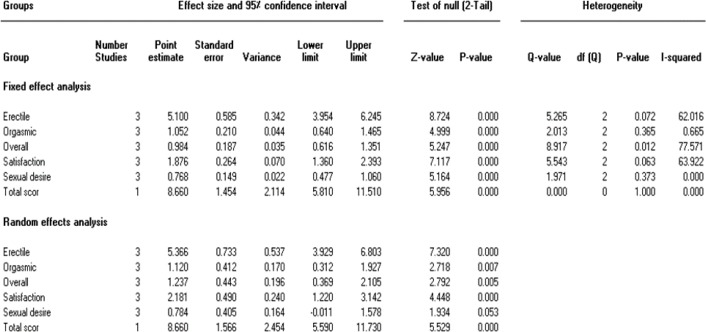
Subgroup analyses of the effects of saffron based on dimensions of Erectile Function questionnaire. SMD, Standardized difference in means; CI, confidence interval

Since the studies were heterogeneous, random effect model was performed and to additionally clarify this heterogeneity, we performed sub-group and sensitive analyses. The results of sub-group analyses according to Sexual desire demonstrated that heterogeneity diminished to 0% in trials. The result of subgroup analysis based on dimensions of Erectile Function questionnaire, show statistically significant differences among subgroups (p=0.00) (see Figure 3).

**Table 2:** Methodological assessment of the quality of the six studies

**No**	Studies	**Criteria used for methodological assessment of the quality of the study **						
		A	B	C	D		E	F
					1	2		
**1**	Shamsa et al.,2009	-	-	+	+	-	+	+
**2**	Heidary et al., 2008	-	-	+	+	-	+	+
**3**	Modabbernia et al., (2012)	+	+	+	+ less than 20%	+	+	+
**4**	Safarinejad et al.,(2010)	+	+	+	-	Not mentioned	+	+
**5**	Safarinejad et al,.(2011)	+	+	+	+ less than 20%	+	+	+
**6**	Mohammadzadeh-Moghadam et al,. (2015)	+	+	+	+ less than 20%	-	+	+

## Discussion

Our study is a systematic review of the available literature regarding the effects of oral administration of saffron on semen parameters in infertile men and to the best of our knowledge, this is the first systematic review and meta-analysis evaluating the impact of saffron on erectile dysfunction. Overall, saffron seems to exert valuable impacts on erectile dysfunction.

**The impact of saffron on semen parameters**

Overall, one study reported a positive impact on semen parameters in terms of sperm motility in men with idiopathic infertility (Heidary et al., 2008 ▶). The fundamental constituents of saffron are coloring carotenoids, crocin, a bitter picrocrocin and its aroma-inducing chemical, safranal. Moreovr, crocetin is another carotenoid of saffron (Alavizadeh and Hosseinzadeh, 2014 ▶, Rezaee and Hosseinzadeh, 2013). Saffron and its constituents have been shown to possess antioxidant activities in different models of oxidative stress. This effect may attribute to its positive effects on sperm motility or infertility. However, Safarinejad et al (Safarinejad et al., 2011 ▶) did not show positive effects, possibly due to the heterogeneity of the study on saffron and the contribution of psychological factors . To sum up, based on the current findings, no consistent conclusion can be made. So, more investigations with consistent statistical analysis of the effect sizes are required.

**The impact of saffron on erectile dysfunction (International index of erectile function questionnaire (IIEF) and its dimensions)**

Three studies reported useful impacts of saffron on erectile dysfunction in all 5 dimensions of the questionnaire namely, erectile function, sexual desire, orgasmic function, intercourse satisfaction, and overall satisfaction (Mohammadzadeh-Moghadam et al., 2015 ▶, Modabbernia et al., 2012 ▶, Safarinejad et al., 2010 ▶). Shamsa's study did not report any numerical data (Shamsa et al., 2009). The mechanism of action of saffron on sexual function is unknown. Both nitric oxide and opioid systems play a noteworthy role in the erectile function (Andersson, 2011 ▶), and saffron seems to interact with both (Khori et al., 2012 ▶, Hosseinzadeh and Jahanian, 2010 ▶). Further, saffron anti-inflammatory, radical-scavenging and neuro-protective properties have been reported in some studies (Hosseinzadeh and Younesi, 2002 ▶, Hosseinzadeh et al., 2012 ▶) . Despite deep-rooted attention to alternative therapy of erectile dysfunction, the mechanism of action and advantages of every treatment option must be completely investigated before its usage (Burnett, 2012 ▶). The all-encompassing perspective of Iranian medicine justifies (Mollazadeh et al., 2015 ▶, Hosseinzadeh and Nassiri Asl, 2013 ▶) the impact of saffron on erectile dysfunction via its capacity to strengthen the heart and enhancement libido, warmth, and vasoconstriction (Sina, 2005 ▶, Tonkaboni, 2011 ▶) . However, these basic outcomes emphasize the need for future studies to clarify the mechanism through which saffron influences erectile dysfunction from the points of view of both Iranian medicine and modern pharmacology.

**Limitations of this meta-analysis**

We detailed the outcomes utilizing random effects model and a rather large heterogeneity was obvious among trials. This heterogeneity can be ascribed to saffron bioavailability, variability among participants, the amount of administered saffron, infertility and erectile dysfunction status, and inconstancy of saffron received from other food-sourced. Also, the quality of almost all of the included studies was not optimal which may decrease the reliability of our results. Future trials ought to be planned in the CONSORT guidelines to improve the quality. 

## Conclusion

Saffron has a positive effect in men with erectile dysfunction. Our results, however, showed contradictory effects of oral saffron on semen analysis in infertile men. Thus, the interpretation of results of the current study is restricted because of methodological flaws and huge heterogeneity among the included studies, and infertility and erectile dysfunction status. Further trials should be done to affirm the present findings.

## Conflict of interest

The authors declare no conflicts of interest. The authors alone are responsible for the content and writing of the paper.
